# Management of Arteriovenous Fistula After Successful Kidney Transplantation in Long-Term Follow-Up

**DOI:** 10.3389/ti.2024.12841

**Published:** 2024-08-12

**Authors:** Jana Janeckova, Petr Bachleda, Petr Utikal, Jirir Orsag

**Affiliations:** ^1^ 2nd Department of Surgery, University Hospital Olomouc, Olomouc, Czechia; ^2^ Faculty of Medicine, Palacký University in Olomouc, Olomouc, Czechia; ^3^ 3rd Department of Internal Medicine, University Hospital Olomouc, Olomouc, Czechia

**Keywords:** AVF flow reduction, AVF ligation, kideny transplantation, screening, hyperfunctional AVF

## Abstract

Arteriovenous fistula (AVF) is the best method of vascular access for hemodialysis. This approach can lead to several complications, such as hyperkinetic heart failure due to a hyperfunctional AVF or dilatation of the feeding artery. These are late complications, especially in patients after a successful kidney transplantation. An observational study was performed focusing on patients more than 12 months after kidney transplantation. The AVF was evaluated by ultrasound and, if the outflow exceeded 1.5 L/min, an echocardiogram was performed. Surgical management was indicated if the cardiac index was higher than 3.9 L/min/m^2^ or upon finding a brachial artery aneurysm. A total of 208 post- kidney transplantation patients were examined over a 3-year period, of which 46 subjects (22.11%) had hyperfunctional AVF and 34 cases (16.34%) of feeding artery dilatation were determined. In total, 40 AVF flow reduction and 6 AVF ligation procedures were performed. The median AVF flow before and after the reduction was 2955 mL/min and 1060 mL/min, respectively. Primary patency after flow reduction was 88.3% at 12 months. Late AVF complications in patients following kidney transplantation are quite common. It is necessary to create a screening program to monitor AVFs in these patients.

## Graphical Abstract

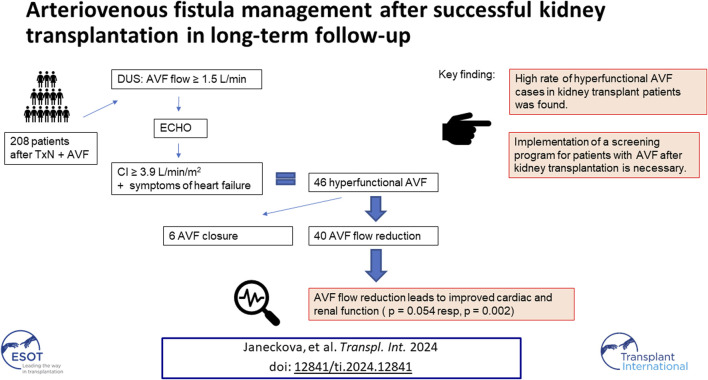

## Introduction

Kidney transplantation is superior to other forms of renal replacement therapy in end-stage kidney disease (ESKD) patients in terms of overall survival and improvement in quality of life [1]. The superior results are achieved by kidney transplantation in the preemptive stage. Despite the slowly increasing number of living donors, most ESKD patients undergo hemodialysis or peritoneal dialysis while waiting for a suitable donor. Arteriovenous fistula (AVF) is the first-line method of connecting a patient to a hemodialysis machine. It is associated with the lowest complication rate when compared to other vascular accesses [2]. Nevertheless, even this vascular access can lead to several complications. Late complications include hyperkinetic cardiac failure due to hyperfunctional AVF or dilatation of the feeding artery, which puts the patient at risk of distal embolism. These late complications also threaten patients after a successful transplantation. Cardiovascular disease is a leading cause of mortality in kidney transplant patients.

After the creation of an AVF, a so-called systemic shunt is formed in the body and the sympathetic nervous system is activated. Several alterations, e.g., cardiac output increase, are immediate, while others develop over time [3]. Left ventricular hypertrophy (LVH), associated with concentric or eccentric remodeling, and dilatation of the left atrium with or without systolic dysfunction develops [4]. The prevalence of LVH in kidney transplant patients remains high, despite the clear benefit of transplantation [5]. Both volume and pressure overload are implicated in the development of LVH. Left ventricular volume overload leads to increased cardiac output (CO). Other factors relevant to LV volume overload are anemia, cyclic hyperhydration and AVF flow. Persistent patent AVF contributes to increased LVH [6]. There is also dilatation of both the feeding artery and the draining vein.

The decision for further management of a functional AVF after successful transplantation remains difficult [7, 8]. In addition to cosmetic aspects, the patient is most at risk for a hyperfunctional AVF, steal syndrome, bleeding and infection. The decision on whether to maintain or ligate the AVF is influenced by the patient’s age, AVF flow, ejection fraction and cardiac output.

There is no clear-cut definition of high-flow AVF. The Vascular Access Society defines high AVF flow as 1–1.5 L/min or 20% of cardiac output. Other authors use a threshold of 2 L/min as high-flow AVF [9, 10].

Retaining the AVF gives the patient a chance to maintain vascular access for hemodialysis after kidney transplant failure. Published literature clearly shows that 20%–50% of AVFs will disappear within the first year after transplantation [11, 12]. The long-term AVF patency rate is no more than 55% [13]. However, the remaining 45% of patent AVFs may be hyperfunctional and threaten the patient due to their “cardiotoxicity.” Deterioration of the transplanted kidney function has been reported after AVF closure [14]. On the other hand, the effect of AVF ligation or flow reduction on LVH has also been reported [9, 15]. Therefore, these procedures are considered justified.

There is no widely accepted screening program for AVF after transplantation. In 2018 we started an observational study with a focus on AVF after kidney transplantation. Due to the high incidence of ultrasound-defined high-flow AVFs, we expanded the study protocol to include an echocardiographic examination when the established threshold AVF flow rate or signs of cardiac insufficiency were exceeded. The observational study became an interventional study focusing on late complications of AVF in patients after kidney transplantation.

## Materials and Methods

The study included patients who were at least 12 months post kidney transplantation, had an AVF prior to the transplant procedure, and had at least 3 successful cannulations for hemodialysis. The baseline inclusion criteria did not specify whether the AVF was functional.

Patients underwent a doppler ultrasound (DUS) examination at the consultation center for vascular access. The brachial artery diameter and its AVF flow were measured. An echocardiographic examination was added when AVF flow was greater than 1.5 L/min. A CO value of 6 L/min, a cardiac index (CI) of 3.9 L/min/m^2^ and symptoms of heart failure were defined as the threshold for the diagnosis of hyperfunctional AVF. Demographic data, renal function, type of immunosuppressive therapy, time since transplantation, time since AVF creation and AVF type were also recorded. Brachial artery aneurysm was defined as a diameter greater than 1 cm and/or the presence of mural thrombi. A simple dilatation of the feeding artery greater than 1 cm in diameter was evaluated as a supply artery dilatation. The diameter of the brachial artery, type of immunosuppression and eventual detection of feeding artery dilatation and aneurysms were part of a previously published paper [16].

When a high AVF flow rate of more than 1.5 L/min was observed, and suprathreshold CO/CI values and symptoms of heart failure were detected, surgery was indicated, namely AVF flow reduction or AVF ligation. Patient preference, history of previous vascular access for dialysis and its complications, function of the transplanted kidney, and time since transplantation influenced the selected surgical procedure. AVF ligation was indicated in cases with very high CO and problematic local findings, in which case the new AVF reconstruction had to be performed using a long expanded polytetrafluoroethylene (ePTFE) prosthesis. For example, a brachiobasilic AVF without transposition of the outflow vein was performed in the past, resulting in a very short cannulation segment.

### Flow Reduction Technique

**FIGURE 1:**
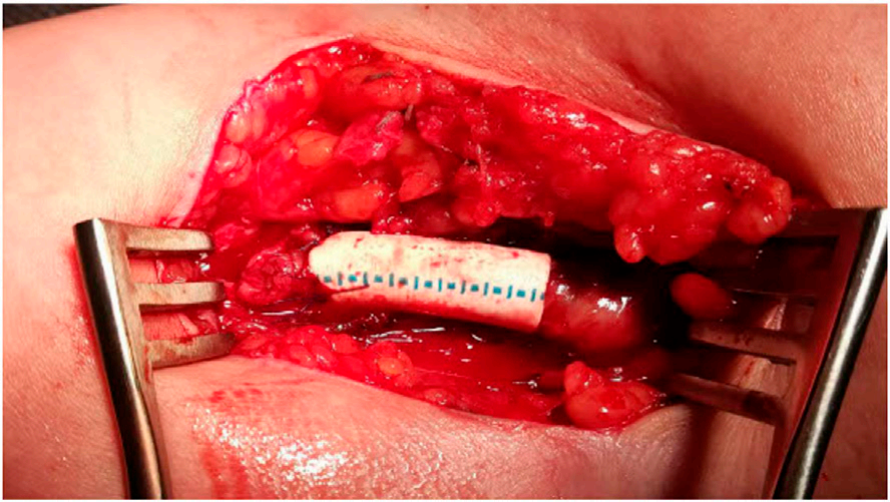
Flow reduction technique: aneurysmorrhaphy with ePTFE prosthesis.

The patients were operated on by two experienced vascular surgeons. The procedure was performed under a regional anesthetic block with antibiotic coverage. After AVF anastomosis, a draining vein with a minimum length of 5 cm was dissected. In the case of a draining vein aneurysm, the entire aneurysm was dissected to the required length. The original anastomosis was resected after heparin administration (2,500–5000 IU) and staple positioning. The excess draining vein wall with aneurysm was resected using Hegar’s dilator and sutured in the sense of aneurysmorraphy. An ePTFE prosthesis with a diameter of 6 mm and a length of approximately 2 cm was then externally attached to the draining vein at the anastomosis (Figure 1). Depending on the local conditions, a new anastomosis was sutured to the artery more distally or the original anastomosis was reduced to a length of 4 mm. If the described technique could not be performed due to the wall thickness of the draining vein or other local findings, the draining vein was resected and a short ePTFE prosthesis was interposed.

Beginning in July 2023, we started measuring the supply artery flow intraoperatively using transit time flow measurement (TTFM) probes before and after flow reduction. After completion of the procedure, drainage is performed, the surgical wound is sutured in layers and a padded bandage is applied. The patient is administered 3 doses of broad-spectrum antibiotics, and after extraction of the drain on the 1st postoperative day the patient is discharged on postoperative days 2 or 3. The first DUS control takes place 4 weeks after the procedure, the next one 5 months after the procedure, followed by further DUS evaluations at 6-month intervals. A follow-up echocardiographic examination is performed 6 weeks after the surgery. Renal function after flow reduction was assessed the first next scheduled post-transplant follow-up visit.

### Ligation of the AVF

The procedure was performed under local anesthesia with antibiotic coverage. After anastomosis dissection, the draining vein at the anastomosis was transected and the original anastomosis was sutured. A DUS control was performed 6 weeks after the procedure. Further ultrasound examinations were performed at 6-month intervals to evaluate the size of the brachial artery.

### Statistics

IBM SPSS Statistics version 22 statistical software was used to analyze the data. A significance level of 0.05 was implemented and a hazard curve was evaluated with a 95% confidence interval. The patency of the reconstructions was evaluated using the Kaplan-Meier curve. Subject data in the monitored groups were anonymized. Spearman’s correlation analysis was used to statistically evaluate the change in CI and CO before and after flow reduction. The Wilcoxon paired test was used to compare paired data.

## Results

A total of 208 kidney transplant patients were examined by DUS from 2018 to 2023. Of the total patient group, 106 functional AVFs (51%) were detected at the initial examination. 46 hyperfunctional AVF cases (43.4% functional AVFs, 22.11% overall) and 34 feeding artery dilatation cases (32.1% functional AVFs, 16.34% overall) were detected, of which 9 were brachial artery aneurysms.

An AVF flow reduction procedure was performed in 40 patients in the study and 6 patients had their AVF closed. Patients indicated for AVF closure had a mean CO of 7.3 L/min, a CI of 4.3 L/min/m^2^ and NYHA III. Five of the six patients who had their AVF closed had a brachiobasilic AVF.

The characteristics of the patients indicated for the flow reduction procedure are provided in Table 1.

**TABLE 1:** Characteristics of patients and vascular access.

		Count	Percentage
sex	Female patients	24	52.2
	Male patients	12	47.8
age	60.2 (36–86)		
time since AVF creation/years	6.0 (1–20; median 4.0)		
time since Tx/years	6.5 (0–25; median 6.8)		
vascular access for dialysis	radiocephalic AVF	13	28.2
	brachiocephalic AVF	22	47.8
	brachiobasilic AVF	11	23.9
cause of ESKD	glomerular disease	9	19.6
	polycystic kidney disease	8	17.4
	interstitial disease	16	34.8
	diabetic nephropathy	8	17.4
	others	5	10.9
immunosuppressive therapy	cyclosporin	4	
	corticosteroids	39	
	mycophenolate mofetil	36	
	tacrolimus	28	
	basiliximab	13	
	everolimus	3	
other comorbidities	peripheral vascular disease	4	8.7
	coronary artery disease	6	13
	diabetes mellitus (not as a primary kidney disease)	7	15.2

TX, transplant procedure.

Aneurysmorrhaphy with external ePTFE prosthesis support was performed in 30 patients (75%). A short ePTFE interposition was inserted after anastomosis resection in 10 patients (25%).

**FIGURE 2:**
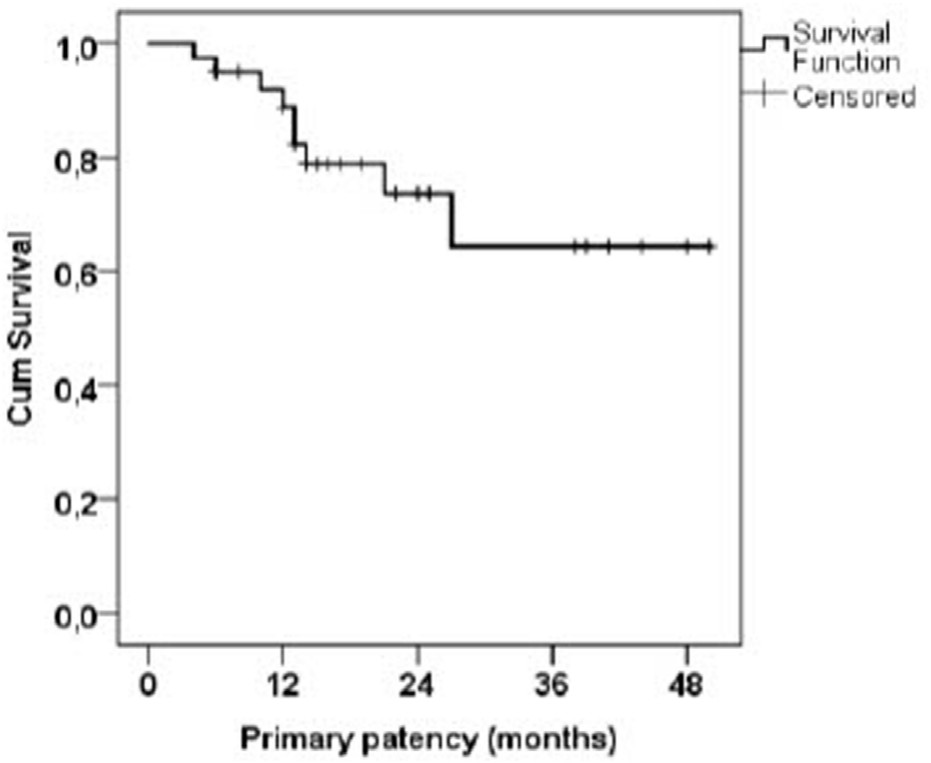
Kaplan-Meier curve of AVF primary patency after flow reduction.

The average AVF flow before flow reduction was 2982 mL/min, with a median of 2918 mL/min, and a range of 1531–5490 mL/min. The average flow 6 weeks after flow reduction was 1126 mL/min, with a median of 1098 mL/min, and a range of 377–1859 mL/min. The primary patency 6 months after the procedure was 95.0% (88.2%–100% with 95%CI), 88.9% at 12 months (78.5%–99.2% with 95%CI), 64.4% at 36 months (42.2%–86.6% with 95% CI); the Kaplan-Meier curve is shown in Figure 2.

Relief of dyspnea and improved performance were reported by 36 patients (90%) at the first outpatient check-up 4 weeks after surgery. A reduction in NYHA classification was found and was statistically significant (<0.0001) after flow reduction.

One patient underwent percutaneous balloon angioplasty of the AVF-reduced anastomosis 36 months after the procedure with good results. One patient underwent flow reduction 11 months after a kidney transplant for high-flow AVF with dyspnea, NYHA III and CO more than 10 L/min. In total, 10 patients completed the follow-up visit 48 months after the procedure. AVF obliteration occurred in two patients. A further 5 patients returned to regular hemodialysis treatment via AVF after flow reduction.

Table 2 lists the parameters considered as possible risk factors for primary patency reduction. None of the monitored parameters is a significant predictor of primary patency duration. No significant difference in primary patency duration was found between the individual types of reduction (external support vs. ePTFE interposition). Table 3 shows the development of cardiac function and renal function before and after flow reduction. An improvement in renal function (serum creatinine and glomerular filtration rate) was observed after flow reduction. A significant decrease in the serum creatinine level and an increase in glomerular filtration rate were demonstrated, p = 0.0002 resp. <0.0001.

**TABLE 2:** Possible risk factors affecting primary patency.

	p-value	RR	95.0% CI for RR	
			Lower	Upper
Age	0.259	0.968	0.914	1.024
time since AVF creation (in years)	0.707	0.969	0.821	1.143
reduction I	0.809	1.219	0.245	6.056
time since Tx/year	0.654	1.020	0.935	1.112
vascular access (1 = reference)	0.639			
vascular access 2 vs. ref.	0.731	0.768	0.171	3.445
vascular access 3 vs. ref.	0.541	1.750	0.290	10.554
Flow before correction	0.970	1.000	0.999	1.001
Sex m	0.503	1.573	0.417	5.929

AVF, arteriovenous fistula.

Vascular access: 1, Radiocephalic AVF; 2, Brachiocephalic AVF; 3, Brachiobasilic AVF; m, male; I, ePTFE, interposition.

**TABLE 3:** Results of AVF flow reduction in 40 patients.

	Before surgery	After	p-value
CO (L/min)	6.51 (5.4–10)	5.72 (3.9–6.61)	0.078
CI (m^2^/L/min)	4.24 (3.9–5,3)	2.99 (2.4–4.5)	0.054
NYHA gr.III (n)	36	4	<0.0001
serum creatinine (umol/L)	163 (83–201)	149 (76–188)	0.0002
GF mL/s/1,73m^2^	0.47 (0.42–1.29)	0.76 (0.44–1.38)	<0.0001

Perioperative flow directly measured with the TTFM probe was 375 mL/min on average (range of 278–409 mL/min), corresponding to a two-fold increase based on indirect ultrasound flow measurement at the brachial artery at 6 weeks.

Infection of the ePTFE cuff developed in 4 patients; there were no cases of early infection of the ePTFE replacement. Almost identical infections occurred in all patients 12-13 months after the flow reduction procedures. All patients had a history of trauma to the affected limb, followed by a brief vascular graft infection complication.

Statistical analysis revealed a positive correlation between the minimum flow and brachial artery size (r = 0.509). Flow reduction was positively correlated with the change in CI (difference before-after), with a correlation coefficient of r = 0.490. The p-value was slightly above the significance level (p = 0.054).

AVF closure was indicated in 6 patients in the monitored group (2.9%). These patients had a dilated AVF feeding artery and very good function of the transplanted kidney. Brachial artery diameter decreased after AVF closure by a median of 4 mm (range 2–8 mm).

Nine cases of brachial artery aneurysm were managed surgically during the monitored period, with a primary reconstruction patency in 87.5% of cases after 12 months. One patient developed an infection of the ePTFE prosthesis, followed by an infection of the basilic vein acquired from the other limb. After the removal of the vascular grafts, the limb was free of ischemia with a patent deep brachial artery.

**FIGURE 3:**
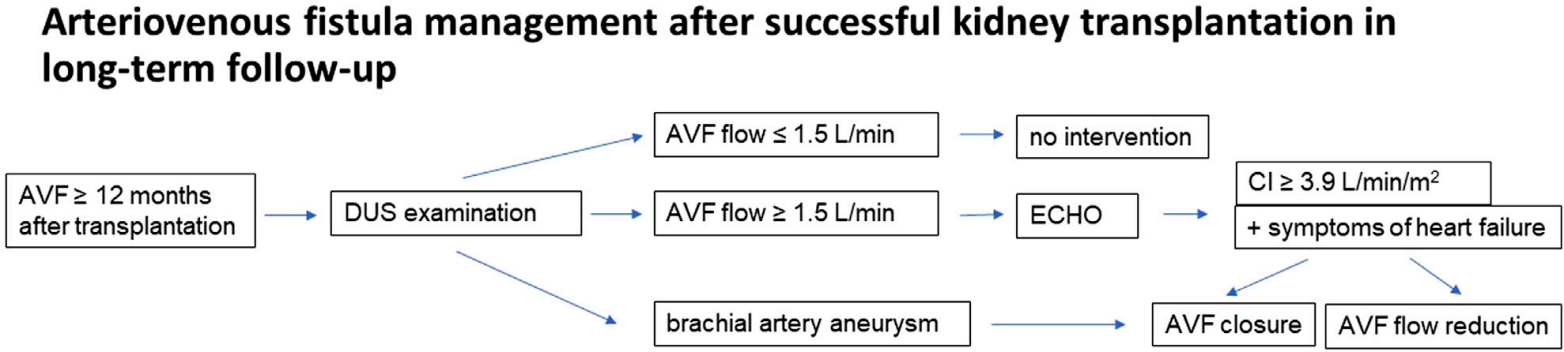
Patient flow chart.

Based on the findings of the study, a methodology for monitoring vascular access has been proposed. During hospitalization after a successful transplant, patients are advised about the need for a follow-up visit at the consultation center for vascular access for an ultrasound examination of the AVF 12 months after the index procedure. This examination is recommended even in the event of vascular access closure within the 1 year. The next follow-up ultrasound examination depends on the outcomes of the first brachial artery size and AVF flow evaluation. If AVF flow is greater than 1.5 L/min, an echocardiographic examination is added. AVF retention, reduction or removal is then considered depending on the cardiac index, brachial artery size and AVF flow. The management process is shown in Figure 3. In addition to the established protocol, patients with clinical problems in the AVF area, dyspnea or hypertension resistant to conservative therapy with a functional AVF are referred for evaluation to the consultation center for vascular access.

## Discussion

Despite the clear benefits of a functional AVF, there are several long-term risks associated with it. This is especially true for patients after a successful kidney transplantation. The decision for further AVF management must be individualized, taking into account the history of vascular access for dialysis, the performance of the transplanted kidney and cardiac symptoms. In the past, the only options were preserving or closing the AVF. With a median graft function of 10.8 years and an average kidney recipient age of 42 years, one-third of transplant patients require dialysis again within 5 years [12]. A retained AVF facilitates this return. Therefore, some authors warn against the ligation of asymptomatic AVFs after transplant [17]. Furthermore, cases of functional deterioration of the transplanted kidney after AVF closure have been published [18], which is why many centers choose to retain AVFs. The cardiotoxicity of a hyperfunctional AVF must be considered in these patients [19]. There is a large study about the hemodialysis access profile in failed kidney transplant patients from the Catalan Renal Registry. It shows, that the main type of vascular access when returning to hemodialysis for failed patients is AVF. In this study, the patients with AVF at the time of kidney transplant showed greater kidney transplant survival compared to those using a catheter. This study is observational, without any information on AVF flow or cardiac function [20]. However, it shows the importance of AVF preservation after a kidney transplant.

Preservation of a functional AVF by aneurysmorrhaphy with external ePTFE support has proven to be an effective and functional option. The borderline statistical significance of the reduction in the decrease in the cardiac index is limited by the small number of patients. We also use this method successfully with non-transplanted patients. The procedure is relatively simple, on a small scale, and can be performed in regional anesthesia. The most substantial effect reported by patients is the rapid relief of dyspnea, followed by improved cardiac function. Furthermore, we noted an improvement in renal function, with the previously published decrease in GF not observed. There was also a decrease observed in the diameter of the brachial artery. This decrease is rather individual. However, we consider it essential that there is no further increase in the size of the arteria brachialis and, with an average flow rate of approximately 1060 mL/min, the risk of developing a brachial artery aneurysm is 3.04 times lower than if the surgery had not been performed [16]. The flow reduction technique and the consequences of a hyperfunctional AVF have also been published by other authors. Our technique with external support is similar to the technique described by Baláž, but we see no reason to consider the use of the prosthesis in the entire extent of the draining vein due to the increase in the size of the surgical wound and the risk of infection [21]. In 2020 the same author described different results of aneurysmorrhaphy in a review. The primary patency of these reconstructions is about 85%, depending on the technique (stapler or no stapler), decreasing to 74% after 12 months [22]. Our results are comparable and, above all, we have a long-term follow-up. The aneurysmorrhaphy technique with external support was used in only 39% of patients with a hyperfunctional AVF. The surgery was intended for aneurysm management and the other patients only had a dilated draining vein [23]. The report did not provide further information about cardiological follow-up, echocardiography control or results after surgery. The same authors recommend AVF ligation in kidney transplant patients with AVF aneurysms and cardiac overload in agreement with the patient and nephrologist [24].

There is no definite AVF flow level that would be completely safe for the patient. A high-flow AVF is defined as an AVF with a flow rate greater than 2 L/min or an AVF flow greater than 30% of cardiac output [25]. Some authors also base the diagnosis of high-flow AVF on signs of heart failure. Other authors define it as a flow rate greater than 1.5–2 L/min regardless of the presence of heart failure [26]. AVF flow may increase over months and years due to feeding artery and anastomosis remodeling. The AVF should always be considered a systematic shunt leading to a decrease in peripheral vascular resistance, a decrease in systemic arterial pressure and an increase in cardiac output. It increases the metabolic demands of the myocardium and leads to the activation of the sympathetic system [9]. Pulmonary hypertension may also develop, leading to a two-fold increase in mortality [27]. Up to 39% of patients with structural heart changes due to a hyperfunctional AVF may be asymptomatic [9]. The clinical effect of AVF depends on the balance between cardiac reserve and AVF function. High-flow AVF can lead to hyperkinetic heart failure and even cardiac arrest. The relationship between AVF flow and cardiac output is nonlinear. Flows above 2 L/min are associated with a significant increase in cardiac output, with all its consequences [3]. In collaboration with our department, Valeriánová described the effect of AVF flow reduction on the myocardium. It is not clear whether cardiac output is related to brachial artery size [10]. However, we confirmed a size reduction of the arteria brachialis after a flow rate decrease or AVF closure. This effect is beneficial in patients with a thin-walled dilated brachial artery, without the risk of distal embolism, but leads to hyperkinetic cardiac overload. Gkotsis published a minimally invasive AVF flow reduction procedure in transplant patients. The technique is similar, but our follow-up is much longer and also monitors the effect of the surgery on the size of the artery [28]. A reduction in flow is clearly associated with an improvement in patient quality of life. Maintaining a functional AVF is of particular benefit in patients with a history of repeated surgeries, where autologous AVF options are limited.

One of the limitations of our study is the long-term risk of immunosuppressive therapy use in the case of ePTFE prosthesis implantation to reduce flow as a possible source of infection. Although the number of infectious complications in our study was low, this risk cannot be neglected. An extracellular matrix instead of ePTFE material may be considered. This material has been used in two kidney transplant patients to reduce AVF flow. The technique of the reduction is unknown; thrombosis occurred in both patients due to stenosis in the venous anastomosis [29]. An extracellular matrix is associated with a relatively high rate of stenosis complications. Therefore, the risk-benefit ratio of not using an artificial material may not be favorable due to the financial burden and the risk of technical failure. Among our 40 patients, only one underwent percutaneous angioplasty due to the stenosis of the anastomosis 36 months after the procedure.

Our data underline the importance of long-term AVF monitoring after kidney transplantation. With a well-adjusted regimen of ultrasound examinations every 12 months, this is not a time-consuming or economically demanding procedure. Close cooperation between the nephrologist and the vascular surgeon is necessary during this monitoring. Similar to the determination of immunosuppressive therapy and the creation of vascular access for hemodialysis, the decision for further AVF management after kidney transplantation must be individualized and based on interdisciplinary collaboration. The possible late complications of AVF, which may be forgotten with prolonged time after kidney transplantation, should always be kept in mind.

Our study had other limitations. The effect of flow reduction on renal allograft survival at our institution could not be considered. The improvement in renal function was not further investigated and may have been influenced by better patient cooperation. The group of patients with high flow AVF is very variable in age, time from kidney transplant and different types of immunosuppressive therapy.

## Conclusion

Our observational-interventional study demonstrated a high rate of hyperfunctional AVF cases in kidney transplant patients. High AVF flow was associated with an increased cardiac index and heart failure symptoms. Patients indicated for a flow reduction procedure benefited substantially, as evidenced by echocardiographic and renal outcomes. Long-term follow-up confirmed this procedure as a safe approach with good results. It is necessary to consider late AVF complications and to implement a screening program for patients after kidney transplantation. The screening program by ultrasound should be started 12 months after a successful kidney transplant. Echocardiography is crucial in high flow AVF. The decision for AVF flow reduction or AVF ligation should be individualized. AVF preservation is preferred. AVF ligation should be done in cases of very high cardiac index with NYHA III or more and problematic local findings for cannulation for hemodialysis.

## Data Availability

The original contributions presented in the study are included in the article/supplementary material, further inquiries can be directed to the corresponding author.
